# Dr. J. M. Locke’s Report on His Analysis of Delabarre’s, Drs. Allen and Hunter’s Formulas for Continuous Gum Work

**Published:** 1855-10

**Authors:** J. M. Locke

**Affiliations:** Chemist, Cincinnati


### DR. J. M. LOCKE'S REPORT ON HIS ANALYSIS OF DELABARRE'S, DRS. ALLEN AND HUNTER'S FORMULAS FOR CONTINUOUS GUM WORK.

[We give this report as a matter of science—giving as it does much valuable information on the subject of which it treats. The practical dentist can very easily see that which may be made available in the Profession.]

CINCINNATI, May 12th, 1855.

GENTLEMEN:—Previous to entering upon the discussion of the characteristic of the several specimens of porcelain termed "Base" for artificial gums. I will draw your atention to some of the terms which are made use of, as an explanation of such terms will better enable us to express ourselves and prevent useless repetition.

It is desirable, upon entering into the discussion of any objects, to make ourselves acquainted with that substance.

Therefore we will make the following quotations from Knapp's Chemical Technology, vol. 2d, page 219.

"*True Porcelain.*"

"*Nature of the mass.*" The clay wares which are known under this name, and which are manufactured in China, and in all European countries, (with the exception of England), are composed of two essentially different constituents, the one of which is an infusible, plastic white clay, called "kaolin or china clay;" the other an infusible, not plastic material, the so called flux. The latter is almost universally composed of feldspar, with the addition of quartz, chalk and gypsum.

"Kaolin alone, would afford a porous opaque body; the flux, however, softens in the heat of the porcelain kiln, and penetrates as a vitrious matter the whole body of the clay, completely filling all the pores, and covering all the surface, it binds the whole together into a dense impenetrable mass. The translucency of this material, is therefore, due to the clay body being saturated as it were with a mass of glass, as transparent paper is permeated with oil. Under the miscroscope, the two ingredients can be clearly distinguished separate from each other, and the milky mass appears as a transparent ground, mixed with an opaque ingredient, which according to Ehrenberg's observations consists of articulated threads, (i. e. composed of globules arranged in a linear direction one on the other), or little rods which are interwoven and cross each other in all directions."

A proximate analysis is such an analysis, as would exhibit the articles of common parlance which compose the substance. Thus, brick and mortar are the proximate portions of a brick wall.

Ultimate analysis, consists in giving all the principles in an isolated condition. Thus the ultimate analysis of the brick wall would be alumina, iron, oxygen, silica, &c., which enter into the composition of the brick; and lime, silica, &c., which form the mortar.

Base is not here used in its chemical sense, but as a technical term in dentistry, signifying the substance used for artificial gums, the coloring being a separate substance, which is afterwards placed upon the base.

*Report.*

M. W. S. Kendall assisted me in preparing the base upon which I experimented. They were not prepared by myself on account of the limited time which I had for making my examination, nevertheless they were prepared under my superintendence, whilst employed at other parts of the examination. The formulas used were taken from the following sources.

That spoken of as "according to Delabarre's formula," was taken from Fitch's Dental Surgery, second edition, page 471, (published 1835). It reads as follows:

| | |
|---|---|
| Porcelain Paste, | 70 parts. |
| Calcined Gypsum, | 10 " |
| White Sand, | 1-20th the mass. |

What oxide you please, 50 grammes by kilogrammes

Dr. Allen's formula, reads as follows:

| | |
|---|---|
| Silix, | 2 oz. |
| Flint Glass, | 2 oz. |
| Borax, | 1 oz. |
| Asbestos, | 2 drachms. |
| Wedgewood, | 1½ oz. |
| Feldspar, | 2 drachms. |
| Kaolin, | 1 " |

This receipt was taken from a printed article, entitled, *The Schedule*, referred to in these *Letter's Patent*, and making part of the same.

Dr. Hunter's formula is the one published in the Vol. vi. No. XII, of the New York Dental Recorder, 1852, viz., granulated body, take any hard tooth material, fuse the following formula, spar. 3 oz., silax, 1½ oz., kaolin ½ oz., fuse completely, &c. Base take flux 1 oz., asbestos, 2 oz., grind together very fine, completely intermixing. Add granulated body, 1½ oz., and mix with a spatula, &c.

It is a well known and admitted fact, that potters frequently change the formula, from which they prepare their ware.

This results from the different quantitative ultimate analysis of different lots of the same proximate materials, they being guided more by the ultimate analysis of the porcelain, when finished than by the proportion of the proximate principles. But, nevertheless, these proximate principles have some slight influence upon the ware.

I will quote from the formulas of the celebrated manufactories of Sevres in France, in support of the former portion of this statement.

The mixture must consist in a hundred parts of,

58.0 silica.
34.5 alumina.
4.5 lime and
3.0 potash.

| *Table I.* | | | | | *Table II.* | | | | |
|---|---|---|---|---|---|---|---|---|---|
| | CONTAINS. | | | | | CONTAINS. | | | |
| Mixture of 1839. Composed of | Silica, | Alumina, | Lime, | Potash, | Mixture of 1843, | Silioa, | Alumina, | Lime, | Potash, |
| 73 Decanted kao. | 30.69 | 30.66 | 0.73 | 1.75 | 48 Decanted kao. | 30 | 17 | 0.05 | 0.96 |
| 24 Feldspar sand, | 19.27 | 3.40 | | 1.27 | 48 Feldspar sand, | 28 | 17 | 0.53 | 2.01 |
| 65 Chalk, | | | 3.77 | | 7.2 Chalk, | | | 4.00 | |
| | 57.96 | 34.06 | 4.50 | 3.02 | | 58 | 34 | 4.58 | 2.97 |

By referring to this table, it is immediately evident that the receipt for compounding the clay was remarkably different in the years 1839 and 1843. It will also be observed that the specimens of kaolin and feldspar respectively, were remarkably different, there by showing that they are not definite chemical compounds, but mechanical mixture of the ingredients which admit of being varied. With all these changes we notice an almost identical resemblance of the ultimate analysis of the two formulas to the desired proportions.

This clearly proves that the proximate analysis may indicate a total want of identity between two species of ware, which identity would be fully established if we regard the ultimate analysis.

I have reduced the three formulas, which at present occupy our attention down to formulas of per centage, thus,

| *Table III.* | | *Table IV.* | | | *Table V.* | |
|---|---|---|---|---|---|---|
| DR. ALLEN'S FORMULA. | | DR. HUNTER'S FORMULA. | | | DELABARRES FORMULA. | |
| Silica, | 28,070 | 22,222 Flux. | Potassa, | 1,709 | Porcelain Paste, | 72,464 |
| White Glass, | 28,070 | | Silica, | 13,675 | Calcined gypsum, | 10,352 |
| Borax, | 14,035 | | Cal. bor. | 6,834 | White Sand, | 4,141 |
| Wedgewood, | 21,053 | 44,444 | Asbestos, | 44,444 | Oxyde, | 13,043 |
| Asbestos, | 3,509 | 33,333 granula. body. | Spar. | 20,000 | | |
| Spar, | 3,509 | | Silica, | 10,000 | | |
| Kaolin, | 1,754 | | Kaolin, | 3,333 | | |

From the foregoing tables I have arranged the following. Tables VI, VII, and VIII, exhibit the proportions of the approximate and ultimate constituents according to the three formulas, whilst in table IX, I have exhibited the ultimate constituents of the three formulas in such a form, that they can be easily compared, and their resemblance, and difference noted.

*Table VI. According to De La Barre's Formula.*

| The proximate constituents taken from the formulas. | Silica, | Alumina, | Lime. | Magnesia. | Oxide of iron, | Oxide of Lead, | Potash or alkali, | Soda, | Sulphuric acid, | Boracic acid, | Foreign matter, loss, &c. | Temp. at which the base vitri fills. |
|---|---|---|---|---|---|---|---|---|---|---|---|---|
| 72,464 Porclelain paste, | 42.029 | 25.00 | 3.261 | .. | .. | ...... | 2.174 | .. | ...... | ...... | .... | 3761° |
| 10,352 Calcined gypsum, | ...... | ...... | 4.291 | .. | .. | ...... | ...... | .. | 6.061 | ...... | .... | |
| 4,141 White sand, | 4.141 | ... .. | ...... | .. | .. | ...... | ...... | .. | ...... | ...... | .... | |
| 13,043 Red lead, | ...... | ...... | ...... | .. | .. | 13,043 | ...... | .. | ...... | ...... | .... | .... |
| | 46.169 | 25.00 | 7.552 | .. | .. | 13.043 | 2.174 | .. | 6.061 | ...... | .... | .... |

*Table VII. According to Dr. Allen's Formula.*

| | Silica, | Alumina, | Lime, | Magnesia, | Oxide of iron, | Oxide of lead, | Potash or alkali, | Soda, | | Boracic Acid, | Loss, &c. | Temp. at which it fused. |
|---|---|---|---|---|---|---|---|---|---|---|---|---|
| 28,070 Silica, | 28.070 | ......... | ......... | ......... | ......... | ......... | ......... | ......... | ......... | ......... | ......... | |
| 28,07 White glass | 12.435 | ......... | ......... | ......... | ......... | 12.085 | 3.299 | ......... | ......... | ......... | 0.253 | |
| 14,035 Calcined borax, | ......... | ......... | ......... | ......... | ......... | ......... | ......... | 4.491 | ......... | 9.544 | ......... | |
| 21,053 Wedgewood, | 13.998 | 5.474 | 0.219 | 0.032 | 12.88 | ......... | 0.042 | ......... | ......... | ......... | ......... | |
| 3,509 Asbestos, | 2.116 | 0.009 | 0.479 | 0.850 | 0.005 | ......... | ......... | ......... | ......... | ......... | 0.050 | |
| 3,509 Feldspar, | 2.342 | 0.614 | 0.044 | ......... | 0.026 | ......... | 0.421 | ......... | ......... | ......... | 0.062 | 22.64° |
| 1,754 Kaolin, | 0.995 | 0.690 | 0.001 | ......... | ......... | ......... | 0.042 | ......... | ......... | ......... | 0.011 | |
| Total, | 59.956 | 6.787 | 0.743 | 0.882 | 1.319 | 12.085 | 3.802 | 4.491 | ......... | 9.544 | 0.376 | |

*Table VIII. According to Dr. Hunter's Formula.*

| | | Silica, | Alumina, | Lime, | Magnesia, | Oxide of Iron, | Potash or alkali, | Soda, | Sulphu-ric acid, | | Boricic acid, | Loss &c. | Temp. at which it fused. |
|---|---|---|---|---|---|---|---|---|---|---|---|---|---|
| 24.22 Flux. | 1.709 Pottassa, | ......... | ......... | ......... | ......... | ......... | 1.709 | ......... | ......... | ......... | ......... | ......... | |
| | 13.675 Silica, | 13.675 | ......... | ......... | ......... | ......... | ......... | ......... | ......... | ......... | ......... | ......... | |
| | 6,838 Calcined Borax. | ......... | ......... | ......... | ......... | ......... | ......... | 2.188 | 4.650 | ......... | ......... | ......... | |
| | 4.444 Asbestos, | 26.804 | 0.116 | 6.071 | 10.769 | 0.067 | ......... | ......... | ......... | ......... | 0,618 | ......... | |
| 33.33 Granulated body. | 20.0 Spar, | 13.350 | 3.500 | 0.250 | ......... | 0.150 | 2.4000 | ......... | ......... | ......... | ......... | ......... | 2.264° |
| | 10.0 Silica, | 1.000 | ......... | ......... | ......... | ......... | ......... | ......... | ......... | ......... | ......... | ......... | |
| | 33.2 Kaolin. | 1.892 | 1.313 | 0.021 | ......... | ......... | 0.075 | ......... | ......... | ......... | 0.032 | ......... | |
| Total, | | 65.721 | 4.929 | 6.342 | 10.769 | 0.217 | 4.184 | 2.188 | 4.650 | ......... | 0.650 | ......... | |

*Table IX. Ultimate Analysis of Base, according to Debarre, Hunter, and Allen's.*

| | Silica, | Alumina, | Lime, | Magnesia, | Oxide of iron, | Oxide of lead, | Potash or alkali, | Soda, | Sulphu-ric acid, | Boricie acid, | Foreign matter, Loss &c. | | |
|---|---|---|---|---|---|---|---|---|---|---|---|---|---|
| Delabarre, | 46.169 | 25.000 | 7.552 | ......... | ......... | 13,043 | 2.174 | ......... | 6.061 | ......... | ......... | Temp. at | |
| Allen, | 59.956 | 6.787 | 0.953 | 0.882 | 1.219 | 12.085 | 3.802 | 4.491 | ......... | 9.544 | 0.376 | which it | 2.264° |
| Hunter, | 65.721 | 4.929 | 6.342 | 10,769 | 0.217 | ......... | 3.184 | 2.188 | ......... | 4.650 | 0.650 | fuses. | |

But few remarks are needed upon these tables. They are not composed from actual analysis, but by calculation from the proportions of the ingredients, whose analysis have been collected from the best authorities, (see appendix,) but we will speak more fully of this subject hereafter.

The following tables exhibit the per centage of linear contraction of platinum and the three specimens of "Base." In cooling from 1645° Farenheit, to 60° F. the former being the temperature at which the specimens prepared according to Hunter's and Allen's formula become solid in cooling after having been heated to vitrification on softening.

*Table X.*

| The formula by which the "base" was prepared. | Per C. of Linear Contraction. | From. | To | Number of degrees through which it cooled. |
|---|---|---|---|---|
| Delabarres, baked only to 2264° | 0.0481 | 1645 | 60° | 1585° |
| " " " 3761° | 0.0486 | ......... | ...... | ......... |
| Dr. Hunter's baked to 2264° | 0.0774 | ......... | ...... | ......... |
| Dr. Allen's, " " 2264° | 0.0767 | ......... | ...... | ......... |
| Platinum (by calculation.) | 0.0790 | ......... | ...... | ......... |

Having found the per centage of contraction, I was desirous of ascertaining the amount which a platinum plate would be distorted by the difference of contraction of the plate and the gum "Base" upon it. Having measured 10 jaws from 2d molar to 2d molar, I found the mean to be 1.77 inches upon which size jaws the calculation were made and presented in the following table,

*Table XI.*

| From who's formula prepared. | Delabarre not vitrified but baked to 2.273° | Delabarre fused at 3.761° | Allen's formula. | Hunter's formula. |
|---|---|---|---|---|
| Con. of base from 2d molar towards 2d mol. | .00815 | 0.00861 | 0.01278 | .01329 |
| Con. of Platinum plate in same direction, | .014 | .014 | 3.014 | .014 |
| Diff. of con. of base and Platinum plate, | .00585 | .00539 | 0.0122 | .00071 |
| Temperature from which they cooled, | 1645° | 1645° | 1645° | 1645° |
| " to " " " | 60° | 60° | 60° | 60° |

The composition according to Delabarre's was quite plastic when moist, on account of the amount of alumina which it contained, when heated it shrank from the plate. This was also owing to the moist or hydrated alumina, which is remarkable for its great contraction, a secondary effect of the contraction was the leaving of large pores, and when the heat was carried to that height, that the portion that vitrifies arrived at that state, the pores were too large to draw in the vitrious mass, hence it was left a porous mass, the heat required for vitrifaction was 3761° F. being about 300° above the temperature required for the melting of iron.

The general character of the specimens of Drs. Allen and Hunter's compound was so close that the description of one will answer for both. They were less plastic than the preceding, (Delabarre), they were not of the same shade of color, and required 2264° (a little above the melting of gold) to vitrify them, at two different times. I made a specimen of each, in the first experiment after vitrification, the specimen according to Dr. Allen's formula was the whitest, but in the second experiment the reverse was the case.

The temperature in these experiments were taken by Daniel's Pyrometer, for the lower temperatures, using an iron rod which was substituted by a platina rod for the highest temperature.

Although somewhat out of place, I will here remark that it would have been but natural and better to have made an actual analysis of the "bases," upon which the experiments of contraction, &c., were made. The reason of it not having been done, was simply that I was called upon at a time too shortly prior to that at which the report was to be delivered.

*Conclusions.*

The question naturally arises, if the alumina produces such a deleterious effects in Delabarre's compound, why do they succeed in making china containing more alumina; the reasons are that any vessel composed of china paste, shrinks proportionally in every part, thereby becoming only smaller without distortion, as it is not connected to any other substance which does not shrink permanently, the heat can then be carried to such an extent as to render the vitrious mass sufficiently fluid to be taken up into the pores; but were the ordinary porcelain teeth present at such a heating, they would be fused into a mass; this precludes the practicability of Delabarre's method, there are two other difficulties to be encountered. If we had teeth sufficiently hard, the great heat required, would preclude the method; but the difference of contraction between platinum and this compound, is such as to distort the work. The difference being even greater than shown in table XI, as the temperature at which the "base" would commence to assume a hard character, would be considerable above 1645°.

In our trial of Delabarre, we gave it an advantage by using a porcelain paste, which is more easily vitrified, that those of France, which are the paste's he refers to, as he directs the person to procure it from the porcelain works. In my table V. I used the analysis of the Sevies porcelain paste, as it is the best French article.

The difference between the Delabarre and Drs. Hunter and Allen, (for we will consider them collectively), consist in their containing so small a quantity of alumina, that in drying they do not shrink from, and therefore not fit to the platina plate; nor does it leave the mass so porous and render it so difficult for the vitrious matter to penetrate it. They (the Allen and Hunter's compound), only require a practicable heat, their rate of contraction approaches closely to that of platinum, [Table XI.] both of which cases Delabarre's compound does not fulfil.

We have previously spoken of Drs. Allen and Hunter's compounds collectively, our reason for so doing are these. They agree so closely in physical character, they vitrify at the same temperature, and approach one another remarkably close in contraction, &c. We will look for a moment to the principle upon which these "bases" are constructed. Some granular refractory substance not effected by ordinary heat is held into a position by the means of hydrated alumina as a paste or glue, and disseminated through this mass is another or other substances which are vitrified at a medium temperature, and solders together the less vitrifiable particles in the form which they have been slightly held by the alumina, by referring to the ultimate analyses, (Tables VII. and VIII.) you will observe nearly the same per centage of alumina which is used as paste. The quantity of silica, also approaches one another, were Bohemian glass to be used in Dr. Allen's formula, then the quantity of silica would be augmented, and no oxyde of lead would be present, they would then approach each other, still more closely. The large quantity of lime and magnesia in Table VIII, is owing to so much asbestos being introduced for the article which is to be soldered by the vitrious mass.

This part of the mass in Dr. Allen's formula, is the Wedgewood, (which resembles Asbestos, see analysis in appendix), asbestos, alumina, and part of the silica. The quantity of matter usually termed flux, for uniting with the silica, &c., to form the vitrious portion is about equal in the two. In Dr. Allen's formula, we have from the glass oxyde of lead 12.08 potash, 3.30, calcined borax 14.03, and 3.50 of feldspar; making 32.9 per cent. Dr. Hunter's formula contains potassa 1.7, calcined borax 6.84, and feldspar 20.00, total, 28.5 per cent. as in the two formulas, there are different proportions of various fluxes; we are not able to arrive at their exact arithmetic value for forming vitrious mass, on account of the *very small* fractional difference.

*Synopsis.*

In our opinion we have shown that Delabarres formula is impracticable. Drs. Hunter and Allen's are identical in physical characters and proportions, their formation involves precisely the same principle, the only difference being a partial substitution of one article for another.

J. M. LOCKE, Chemist.

